# Efficacy of home-based exercise in the treatment of pain and disability at the hip and knee in patients with osteoarthritis: a systematic review and meta-analysis

**DOI:** 10.1186/s12891-024-07585-w

**Published:** 2024-06-26

**Authors:** Yichen Mao, Boyuan Qiu, Weiwei Wang, Pengwei Zhou, Zhixue Ou

**Affiliations:** 1 Guilin Traditional Chinese Medicine Hospital, No. 2, Lingui Road, Xiangshan District, Guilin City, Guangxi Province 541000 China; 2 Guangxi University of Traditional Chinese Medicine, Nanning, Guangxi 530000 China

**Keywords:** Home-based exercise, Knee osteoarthritis, Hip osteoarthritis, Meta-analysis, Kinesitherapy

## Abstract

**Background:**

An increasing body of evidence suggests that home-based exercise (HBE) therapy has significant therapeutic effects on knee osteoarthritis (KOA) and hip osteoarthritis (HipOA), and it has advantages such as cost savings, strong operability, and good compliance compared with hospitalization and exercise courses.

**Objective:**

To evaluate the efficacy of HBE in the treatment of KOA and HipOA.

**Methods:**

A systematic search was conducted in PubMed, Cochrane, Web of Science, and Embase to collect randomized controlled trials. The retrieval time was from database establishment until March 6, 2024. Stata 15.1 software was used for data analysis.

**Results:**

A total of 16 randomized controlled trials involving 3,015participants were included, with 1,519 participants in the intervention group and 1,496 in the control group. The meta-analysis showed that, compared to the control group, HBE can significantly improve pain [SMD=-0.38, 95% CI (-0.58, -0.18); *P* = 0.001], joint function      [SMD=-0.60, 95% CI (-1.01, -0.19); *P* = 0.004], balance ability [SMD=-0.67, 95% CI (-1.00, -0.34); *P* = 0.001], mobility (ADL) [SMD = 0.51, 95% CI (0.19, 0.82); *P* = 0.002] in patients with KOA and HipOA. There is no statistical difference in the improvement of joint stiffness [WMD = -0.80, 95% CI (-1.61, 0.01); *P* = 0.052]. In addition, subgroup analysis showed that HBE significantly improved pain, joint function, and balance ability in KOA patients compared with the control group. HipOA patients showed significant improvement in pain and joint function; However, HBE only improved activity ability in patients with comorbidities of KOA and HipOA.

**Conclusion:**

HBE can effectively alleviate pain, improve joint function, and enhance physical function in patients with KOA and HipOA. However, more high-quality randomized controlled trials (RCTs) with large sample sizes and long-term interventions are needed to validate the efficacy of HBE due to limitations in the methodology and consistency of indicator outcomes in the included RCTs.

**Registration number:**

We’ve registered with PROSPERO, and the number is CRD42023443085.

**Supplementary Information:**

The online version contains supplementary material available at 10.1186/s12891-024-07585-w.

## Introduction

Osteoarthritis (OA) is a common chronic joint disease characterized by joint pain and morning stiffness. In 2019, it affected 528 million people globally [1]. Currently, there is no effective treatment that has been proven to slow the progression of the disease [2]. The latest estimates from the Global Burden of Diseases, Injuries, and Risk Factors Study (GBD) found that the age-standardized prevalence and incidence of symptomatic, radiographically confirmed OA increased by 9.3% (95% UI 8–10.7%) and 8.2% (95% UI 7.1–9.4%), respectively, from 1990 to 2017 [3, 4]. In the 2019 Global Burden of Musculoskeletal Diseases survey, OA accounted for 20.1% of the demand for musculoskeletal rehabilitation, placing a huge economic and public healthcare burden on individuals, families, and society [5, 6]. Knee osteoarthritis (KOA) and hip osteoarthritis (Hip OA) are the two most common types of osteoarthritis in the lower limbs due to weight-bearing and mobility. The knee joint is the most complex joint in the human body, bearing the greatest load among all joints, making it the most prone to osteoarthritis [7]. Although all factors affecting joints in osteoarthritis have commonalities, unlike KOA, hip OA is often associated with hip dysplasia and acetabular impingement syndrome, two risk factors [8, 9].

Age, obesity, heavy manual labor, and high-intensity exercise are all risk factors for OA. Patients with certain comorbidities have a higher incidence of OA and an increased risk of activity limitations caused by OA, making it more difficult to manage their condition [6, 10]. In addition to surgery, medication, and physical therapy, exercise is considered the cornerstone of OA treatment. The Osteoarthritis Research Society International (OARSI) and American College of Rheumatology (ACR) guidelines recommend that regardless of whether there are comorbidities, diet weight management, regular and sustained exercise plans, and mind-body exercises (such as Tai Chi, yoga) should be regarded as the core therapeutic modalities for OA [11, 12].

Patients with OA may avoid exercise due to pain and fear of exercise-related injury. Shur et al. [13] found that a lack of physical activity can lead to age-related muscle loss and decreased muscle quality, which is unfavorable for the prognosis of patients with KOA [14]. Increasing evidence supports exercise as a maximally effective treatment for alleviating symptoms and related comorbidities of OA [15]. An international consensus study [16] has developed evidence-based recommendations for OA exercise, which include tailoring personalized exercise plans based on patients’ conditions, optimizing modes and dosages, and emphasizing compliance and exercise education. Despite the strong recommendation for exercise therapy in OA guidelines. However, it is difficult to form standardized exercise prescriptions, which are multidimensional and complex, due to insufficient research on clinical controlled trial data, resulting in difficulties in efficacy assessment and comparative research [15, 16]. Hospital-based treatment plans and exercise programs do not confer a long-term prognosis benefit over home-based exercise (HBE) therapy. HBE is an effective way to maintain rehabilitation and combination therapy after discharge. In contrast, HBE therapy offers several advantages, including cost savings, practicality, high compliance, improved comfort during rehabilitation, and reduced risk of injury associated with travel to clinics. Hurley M et al. [17] found that exercise can improve physical function, depression, and pain in patients with KOA and hip OA. Jönsson et al. [18] showed that early-stage and mild KOA/HipOA patients, particularly those who declined surgical intervention, experienced significant relief of clinically relevant pain when they participated in a self-management program that incorporated HBE. Currently, there is no substantial evidence indicating the clinical efficacy and superiority of HBE. Therefore, this meta-analysis aims to address this question and provide clinical physicians and patients with exercise therapy plans, data analysis, and references.

## Methods

### Search strategy

Two independent researchers searched four databases, PubMed, Embase, Cochrane, and Web of Science, using a combination of topic words and free words. The search was conducted from the establishment of the database until March 6, 2024. The search keywords mainly included Knee Osteoarthritis, Knee Arthritis, Hip OA, Coxarthrosis, and Home-based exercise. In addition, the two researchers also reviewed the references of similar studies to ensure the inclusion of relevant literature that was not searched. The detailed search strategy is shown in Supplementary Table 1.

### Inclusion and exclusion criteria

Specific inclusion criteria were as follows: (1) Participants meet one or more of the diagnostic criteria for KOA and HipOA in the Kellgren Lawrence classification (KL scale), ACR, and American Rheumatology Association (ARA), or had written diagnosis or clinical imaging evidence from a doctor to prove the diagnosis of KOA and HipOA [19–22]; (2) The intervention group received HBE, without restrictions on specific forms of exercise; The control group received blank controls, health education and publicity, and non-steroidal anti-inflammatory drugs, excluding exercise interventions; (3) The patient’s gender, age, race, and source of the case were not limited, without any restriction on whether to use a blinding method. Studies had to be published in English; (4) The outcome measures encompassed the Western Ontario and McMaster Universities Osteoarthritis Index (WOMAC), Visual Analogue Scale (VAS), timed up and go test (TUG), timed chair stand (TCS), gait speed (GS), the six-minute walk test (6MWT), the five-times sit-to-stand test (FTSST) [23–25]; (5) The search design was a randomized controlled trial (RCT).

The specific exclusion criteria were as follows: (1) Literature review, meta-analysis, duplicate publications, conference abstracts, animal experiments, case reports, protocols, non-randomized controlled trials and interventions that do not meet the inclusion criteria, and unavailable full-text and data; (2) Patients with a history of knee joint trauma, surgery, or rheumatoid arthritis; (3) duplicate publications.

### Literature screening and data extraction

The literature was screened by two independent reviewers, who read the title, abstract, and full text, extracted data, and cross-validated the findings. Any discrepancies were resolved through discussion with a third reviewer to reach a final decision. Duplicate publications were first automatically searched using Endnote software and then manually reviewed and removed. Titles and abstracts were screened before reading the full texts of selected articles. Subsequently, two evaluators independently extracted related information from the selected studies based on a standardized data extraction table (Table 1). The main extracted information included the first author’s name, publication year, country, sample size, gender, age, intervention measures, treatment period, and outcome measures.

**Table 1:** Basic characteristics of included studies

Study	Year	Country	Sample size		Gender (M/F)	Mean age(years)		Interventional protocol			outcome	Type
			HBEG	CG		HBEG	CG	HBEG	CG	Time of treatment		
Oh, S.L [32]	2021	South Korea	40	20	None	72.44 ± 6.30	71.06 ± 5.41	HBE 2-3times/week HEP 50 min/month	HEP 1 × 50 min/month	20 W	F1,F4,F5,F6,F7,F9	KOA
Krauss, I [33]	2020	Germany	64	63	77/50	57.8 (9.6)	60.3 (8.8)	HBE 2 × 60 min/week	NT	12 W	F9	Hip OA
Chen.H [38]	2019	China	71	70	22/119	68.9(7.78)	68.8(6.96)	HBE 3 × 30 min/week HEP pre-6 weeks 60 min/2 weeks	HEP pre-6 weeks 60 min/2 weeks	12 W	F1,F5,F7,F8,F10	KOA
Steinhilber, B [28]	2017	Germany	70	68	None	58 ± 19	60 ± 9	HBE 2 × 60 min/week	NT	12 W	F3	Hip OA
Takacs, J [27]	2017	Canada	20	20	8/32	66.1 (8.7)	67.1 (5.4)	HBE 4 times/week	NT	10 W	F3,F4	KOA
C.H. Teirlinck [39]	2016	Netherland	101	102	84/119	64 (8.5)	67 (9.6)	HBE 1 × 30 min/week	general practitioner care	12 M	F3,F7	Hip OA
Armagan.O [41]	2015	Turkey	30	40	15/55	55.9 ± 4.9	56.8 ± 3.7	HBE 2 times/week	Glucosamine 1.5 g/d	24 W	F1,F2,F4,F5	KOA
Zeng, R.M [26]	2015	China	32	27	31/28	65.19 ± 2.61	64.81 ± 2.48	HBE 5 × (45 min Tai chi + 30 min cinesiatrics)/week HEP	HEP	12 W	F1,F4,F7,F8	Hip OA
Eun-Lee Lee [31]	2023	Korea	15	16	None	65.63 ± 3.7	68.27 ± 4.78	HBE 3 × 35 min/week	NT	8 W	F2,F7,F10	KOA
Bennell, K.L [40]	2010	Australia	45	44	46/43	64.5 (9.1)	64.6 (7.6)	HBE 5 times/week	NT	12 W	F1,F3,F4	KOA
Doi T [37]	2008	Janpan	63	58	31/90	67.4 ± 13.4	71.2 ± 22.2	HBE 2 × 15 min/day	NSAIDs	8 W	F1,F2	KOA
Hughes, S. L [34]	2006	Chicago	115	100	48/167	73.3	73.4	HBE 3 × 90 min/week	HEP	12 M	F1,F4,F5,F6,F8	KOA, HipOA
Hughes, S.L [35]	2004	Chicago	80	70	125/25	73.5	73.7	HBE 3 × 90 min/week	HEP	8 M	F1,F4,F5,F6,F8	KOA, HipOA
Ravaud, P [30]	2004	France	735	760	424/1071	None	None	HBE 4 × 30 min/week	Usual care	6 M	F1,F2,F4	KOA, HipOA
Evcik, D [36]	2002	Turkey	27	26	17/36	56.3 ± 6.1	55.8 ± 6.9	HBE 2 × 10 min/day	NT	12 W	F1,F2,F4	KOA
Rogind, H [29]	1998	Denmark	11	12	2/21	69.3 _+ 8.2	73.0 _+ 6.5	HBE 1 time/day HEP 2 times/week	NT	12 M	F3,F9	KOA

*Abbreviations* HBEG: home-based excises group; CG: control group; M/F: male/female; HBE: home-based excises; HEP: Health education program; NT: no treatment; F1: WOMAC pain; F2: VAS; F3: Knee pain/Hip pain (11-point NRS); F4: WOMAC function; F5: WOMAC stiffness; F6: TCS(timed chair stand); F7: TUG(timed up& go); F8:6MWT(The six-minute walk test); F9: GS( gait speed); F10: FTSST(The five-times sit-to-stand test)

### Quality assessment

Two independent researchers adopted Cochrane Handbook for Systematic Reviews of Interventions was used to assess the methodological quality of the included studies. The assessment included random sequence generation, allocation sequence concealment, blinding of participants and personnel, blinding of outcome assessment, incomplete outcome data, selective outcome reporting and other bias. Each domain was rated as “low risk of bias,” “high risk of bias,” or “unclear” (lack of relevant information or unclear bias). The evaluation results were confirmed by two researchers after cross checking. The evaluation results of the two researchers were tested for consistency using Kappa. A Kappa value less than 0.2 indicated poor consistency, 0.2–0.4 indicated average consistency, 0.4–0.6 indicated moderate consistency, 0.6–0.8 indicated strong consistency, and 0.8-1.0 indicated strong heterogeneity.

### Statistical analysis

Stata 15.0 was used for meta-analysis, and heterogeneity was tested using Cochran’s Q test and Higgins I² Quantitative statistics. Continuous variables were represented by mean difference (MD), and if the units were inconsistent, standardized mean difference (SMD) was used. The effect size and 95% confidence interval (CI) were used as the statistical measures for evaluating their effects. The I^2^ value was used to test the heterogeneity. If the homogeneity was good (I^2^ < 50%), a fixed-effects model was used. If the heterogeneity was large (I^2^ ≥ 50%), a random-effects model was used. When there was excessive heterogeneity, sensitivity analysis and subgroup analysis were used to explore the sources of heterogeneity. Funnel plots were used to visually reflect publication bias, and Egger’s test was used to statistically test publication bias; *P* > 0.05 indicates the existence of publication bias, and the trim and fill method was used. Further sensitivity analysis was conducted to examine the stability of the research results. The statistical significance of the merged statistics of the included studies was set at *P* < 0.05.

## Results

### Literature screening

**Fig. 1:**
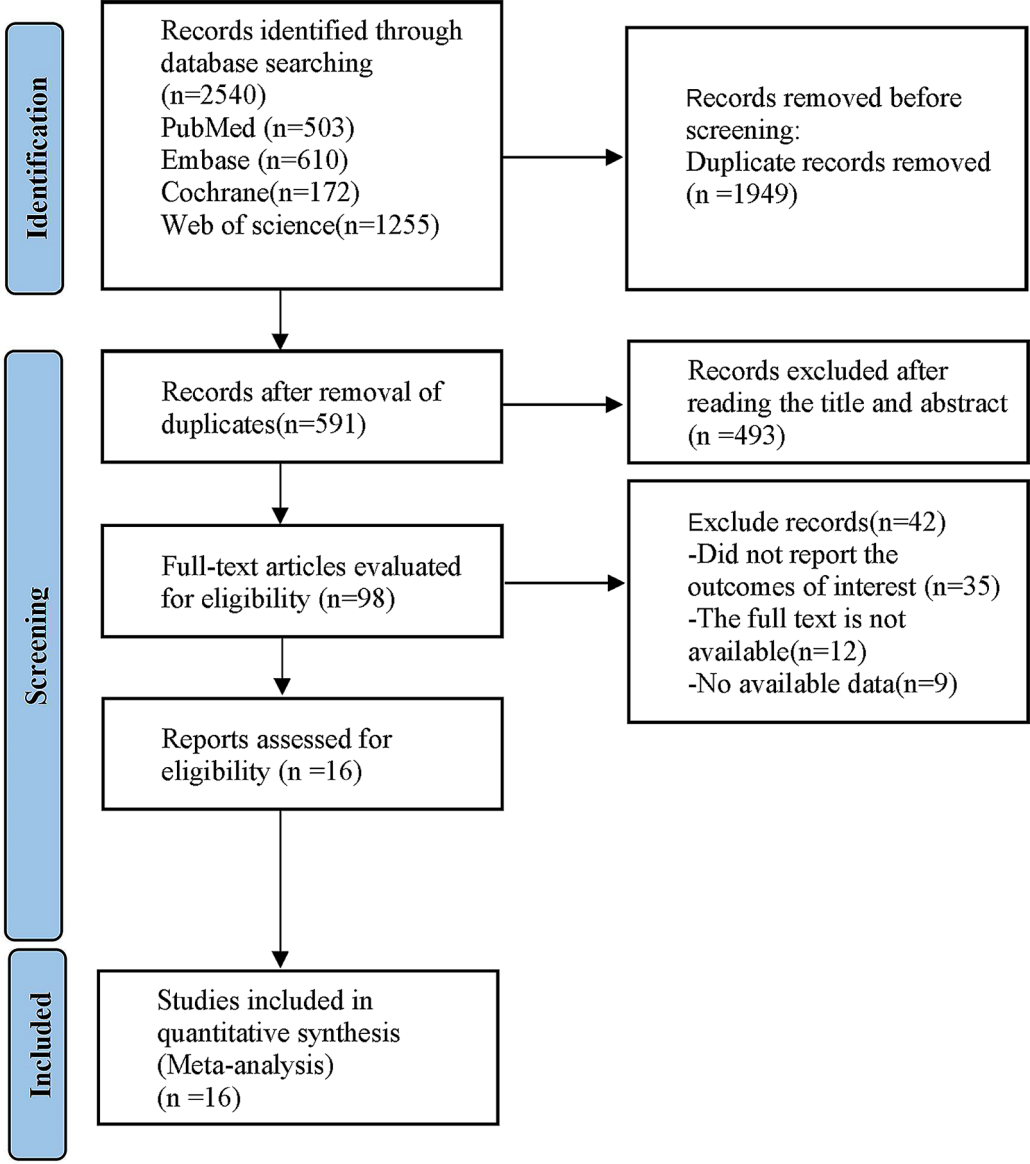
The whole literature selection process

As shown in Fig. 1, a total of 2,540 articles were searched from the four databases, 1,949 of which were duplicate articles or marked as mismatched by automatic tool and were excluded. After reading the title and abstract, 493 articles that clearly did not meet the inclusion criteria were excluded. Subsequently, the full texts of the remaining 98 articles were searched and read. According to the inclusion criteria, 82 articles that did not meet the inclusion criteria were excluded, including reviews, meta-analyses, duplicate publications, conference abstracts, animal experiments, case reports, protocols, non-randomized controlled trials, and intervention measures. Finally, a total of 16 RCTs were included in this meta-analysis.

### Basic characteristics of included studies

A total of 3,015 KOA patients were involved in the 16 finally included studies, all of which were published in English between 1998 and 2023 [26–41]. The countries where the patients were located included China, the United States, South Korea, Germany, Canada, Türkiye, the Netherlands, Australia, Japan, France and Denmark. These patients all meet one or more diagnostic criteria for KOA or HipOA in the ARA, ACR, and KL scale.

The basic information of the participants involved in all 16 studies is as follows: the average age of these participants ranged from 55 to 74 years old; The participants in all 16 study consisted of males and females; Three studies reported the medication history of participants [27, 31, 35], and 5 studies reported the underlying diseases of participants [20, 26, 30, 35, 38]. Regarding intervention methods, the control group were treated with blank controls in 7 articles [26–28, 32, 35, 36, 40], health education lectures in 5 articles [26, 31, 33, 34, 37], routine care in 3 articles [29, 30, 38], and medication in 1 articles [37]. The follow-up time for these studies ranged from 8 to 12 months. Regarding the three main outcomes, one study did not report pain [32], 7 studies did not report joint function-related indicators [28, 29, 31, 33, 37–39], and 11 studies did not report joint stiffness [26–30, 32, 35–39]. The baseline characteristics of the included studies were comparable. The specific characteristics of the included studies are shown in Table 1.

### Risk of bias assessment

**Fig. 2:**
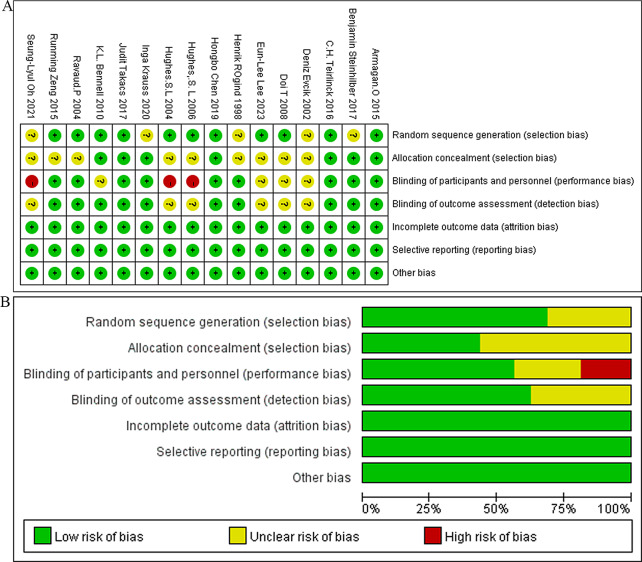
(**A**) Risk of bias summary; (**B**) Risk of bias summary

Based on the Cochrane bias assessment criteria, 16 studies were evaluated, with 11 articles providing a clear description of their specific methods for randomization and 7 articles explaining the blinding methods used. Three studies did not use blinding methods, while 10 studies blinded the outcome evaluators. The risk of bias assessment for the included studies is shown in Fig. 2A-B. Subsequently, Kappa test and paired chi square analysis were performed on the evaluation results of the two researchers. The results showed a Kappa value of 0.73 (*P* = 0.001) and paired chi square analysis *P* = 0.923, indicating good consistency in the evaluation results of the two researchers.

### Meta-analysis results

#### Main outcome measures

**Fig. 3:**
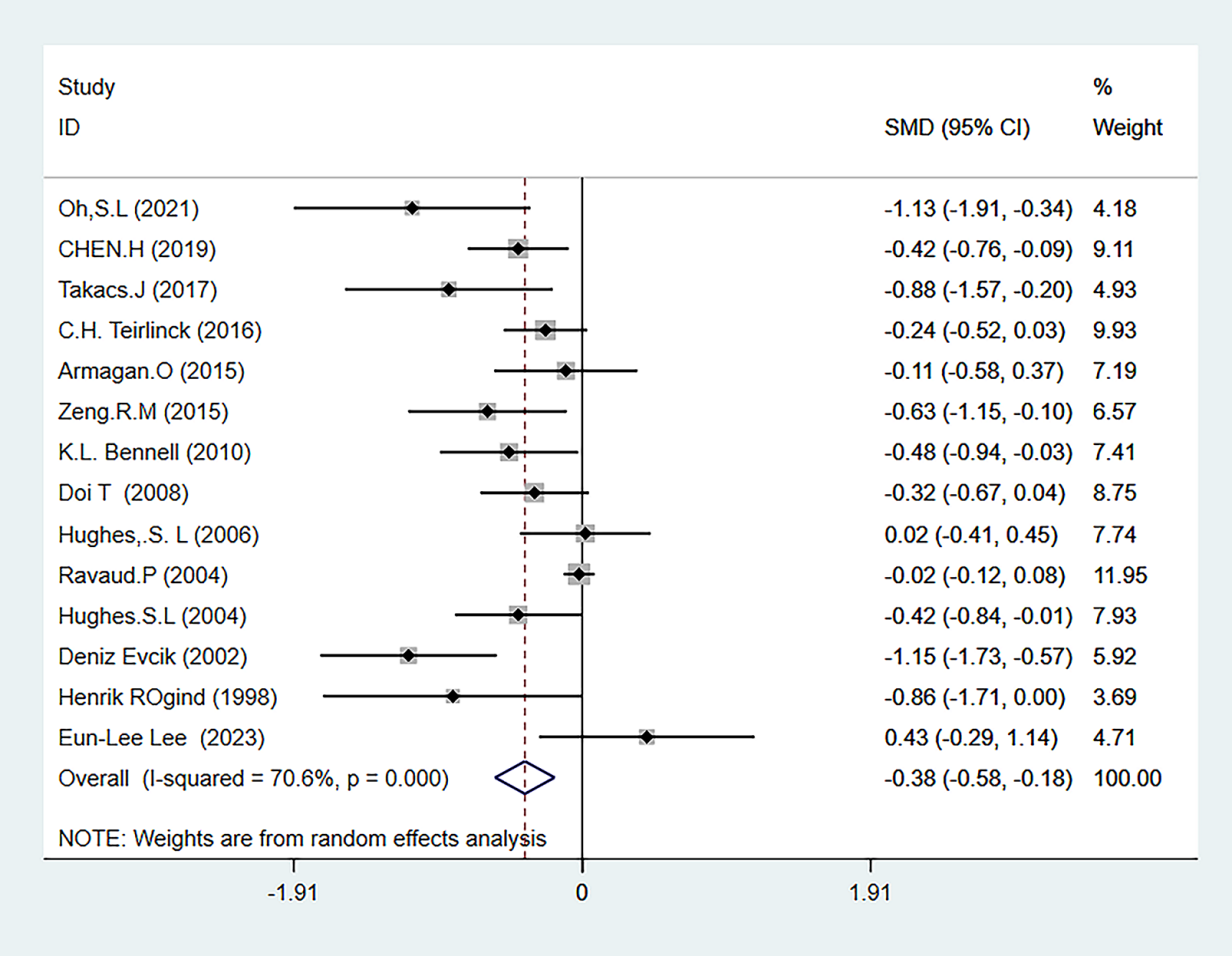
Forest map of pain meta-analysis

The three main outcome measures of this meta-analysis were pain (evaluated using the WOMAC pain subscale, VAS, and NARS 11-point combined assessment), WOMAC stiffness subscale, and joint function (evaluated using the WOAMC function subscale and TCS combined assessment). The results showed that HBE significantly improved pain [SMD=-0.38, 95% CI (-0.58, -0.18); *P* = 0.001] and joint function [SMD=-0.60, 95% CI (-1.01, -0.19); *P* = 0.004] in KOA and HipOA patients, but there was no statistically significant difference in the improvement of joint stiffness [WMD = 0.80, 95% CI (1.61, 0.01); *P* = 0.052]. The main outcome indicators are shown in Fig. 3-Fig. 5, and Table 2. The grade rating of the main outcome measures is shown in Table 3.

**Table 2:** Summary of meta-analysis results

Measurements	Number of included articles	Number of patients involved	I² value (%)	SMD/WMD (95%CI)	*P*-value
Primary outcome measures					
pain	14	2526	70.6	-0.38 (-0.58, -0.18)	0.001
stiffness	5	429	89.8	-0.80 (1.61,0.01)	0.052
function	9	2007	91.3	-0.60 (-1.01, -0.19)	0.004
Secondary outcome measures					
balance	5	608	70.5	-0.67 (-1.00, -0.34)	0.001
ADL	7	66.5	66.5	0.51 (0.19, 0.82)	0.002

##### Pain

A total of 14 articles quantified the level of pain in patients [26, 27, 29–32, 34–41], involving 2,526 participants, with 1,280 in the HBE group and 1,246 in the control group. The meta-analysis showed heterogeneity (I^2^ = 70.6%), so a random-effects model was used to analyze the studies. The analysis results showed that HBE was more effective in reducing pain levels in KOA and HipOA patients than the control group [SMD=-0.38, 95%CI (-0.58, -0.18); *P* = 0.001] (Fig. 3).

##### WOMAC stiffness

**Fig. 4:**
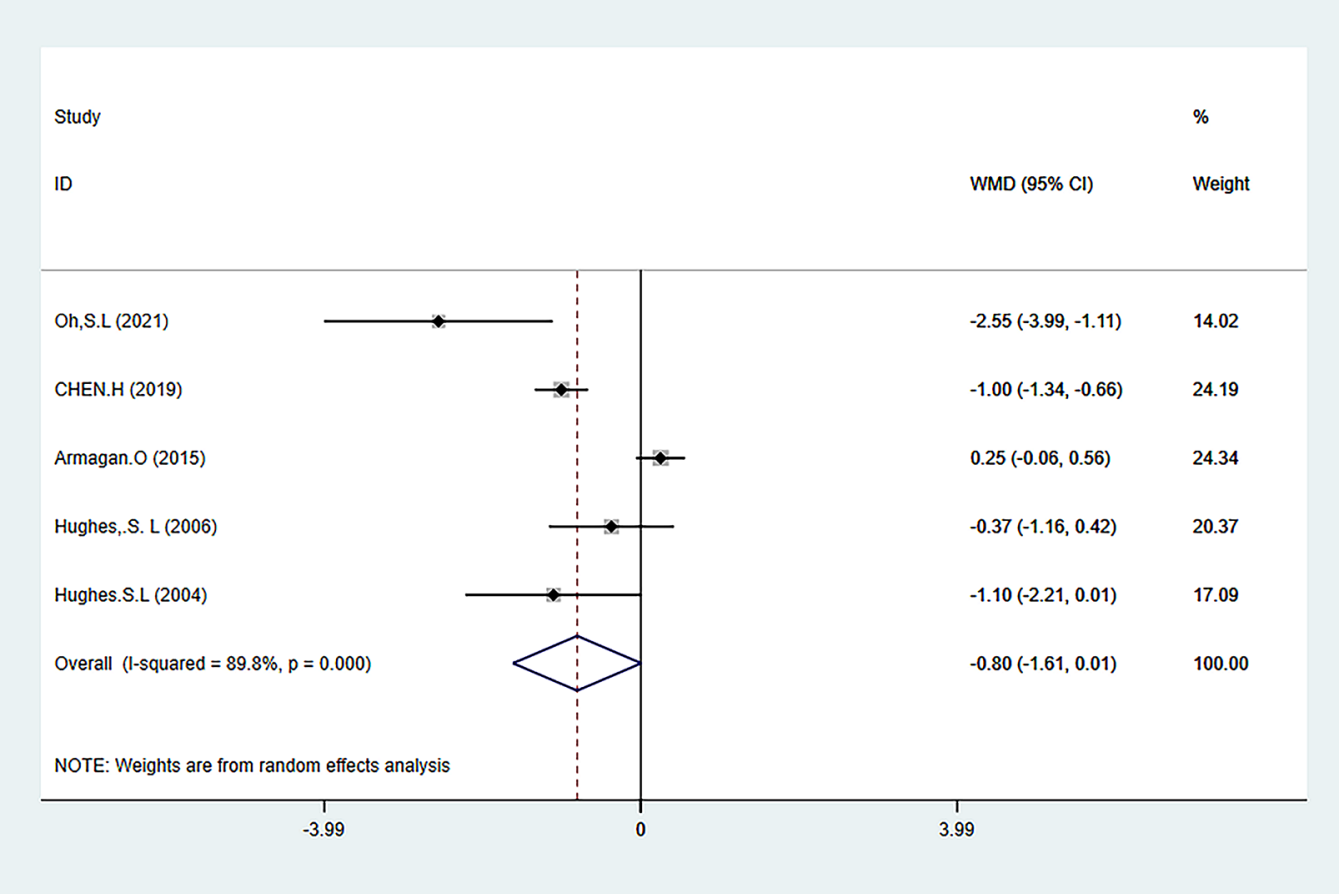
Forest map of meta-analysis of joint stiffness

WOMAC stiffness scores were mentioned in 5 articles [32, 34, 35, 38, 41], involving 429 participants, with 240 in the HBE group and 189 in the control group. The meta-analysis showed heterogeneity (I^2^ = 89.8%), so a random-effects model was used to analyze the studies. The analysis results showed that no significant statistical differences were observed in stiffness levels between the HBE group and the control group [WMD=-0.80, 95% CI (-1.61,0.01); *P* = 0.052] (Fig. 4).

##### Joint function

**Fig. 5:**
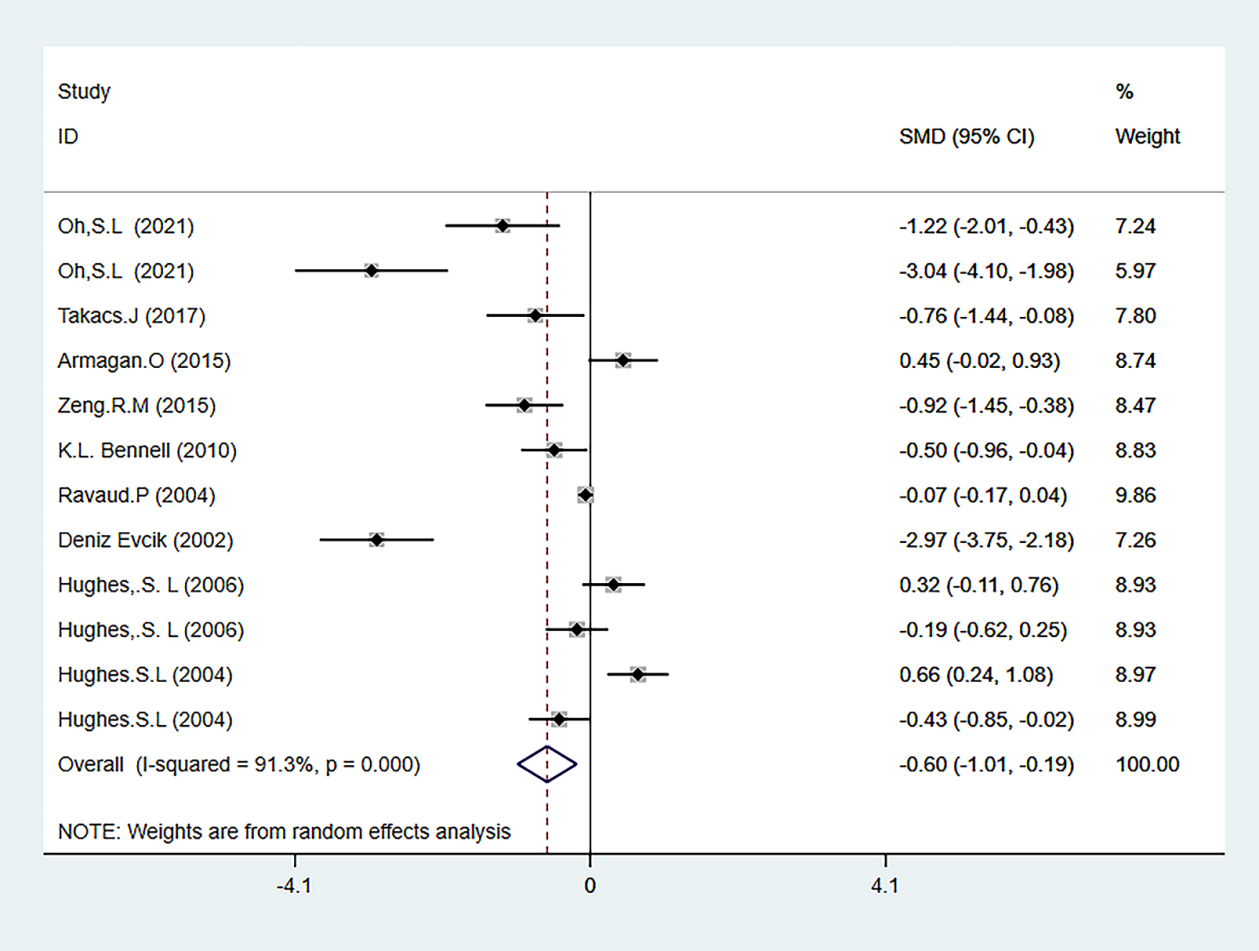
Forest map of meta-analysis of joint function

Joint function was evaluated using two tests, WOAMC function and TCS. A total of 9 articles reported relevant tests [26, 27, 30, 32, 34–36, 40, 41], involving 2007 participants, with 1,019 in the HBE group and 988 in the control group. The meta-analysis showed heterogeneity (I^2^ = 91.3%), so a random-effects model was used to analyze the studies. The analysis results showed that HBE was more effective in improving joint function in KOA and HipOA patients than the control group [SMD=-0.60, 95% CI (-1.01, -0.19); *P* = 0.004] (Fig. 5).

#### Meta-analysis of the secondary outcome measures

**Fig. 6:**
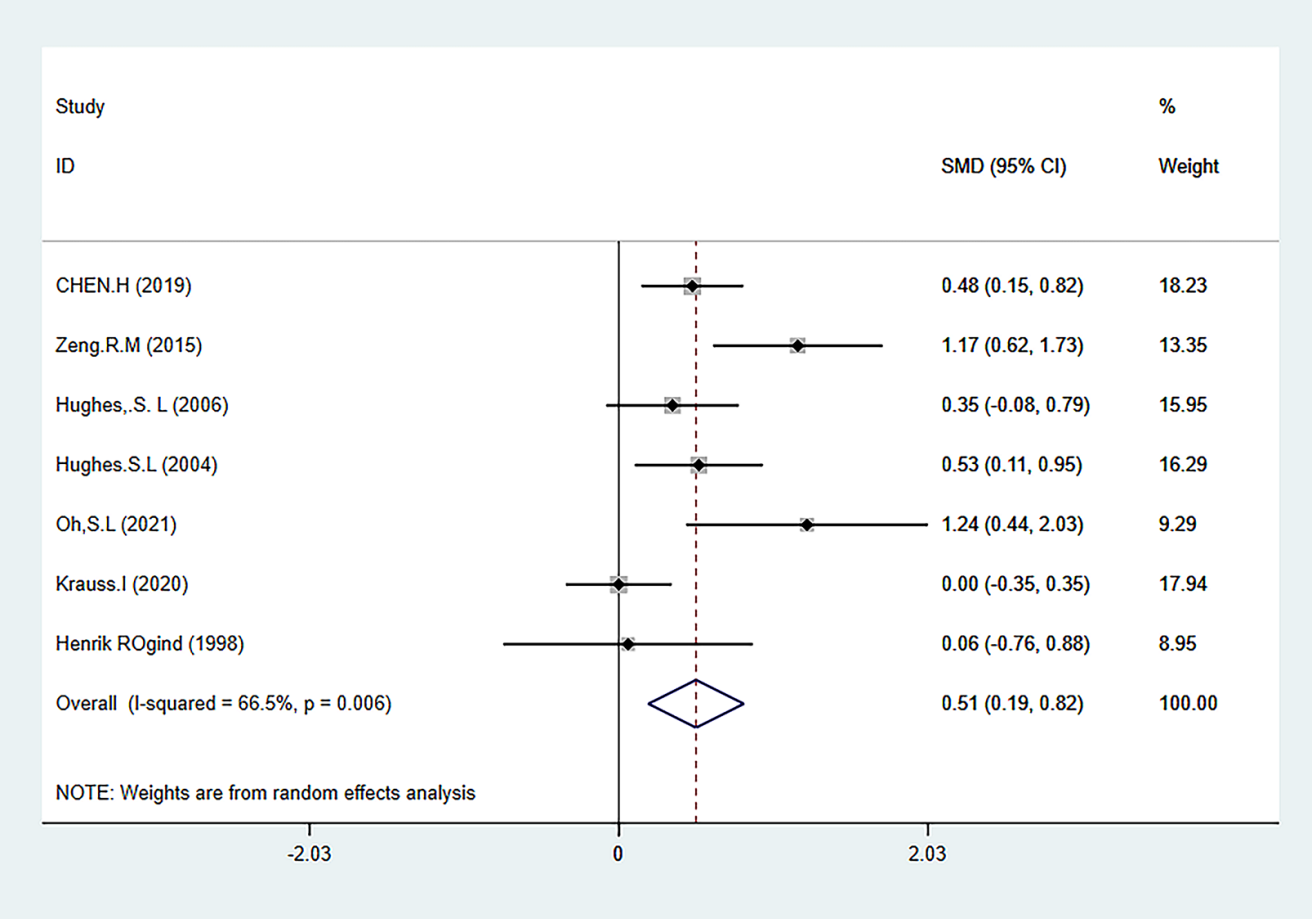
Forest map of activity capability meta-analysis

**Fig. 7:**
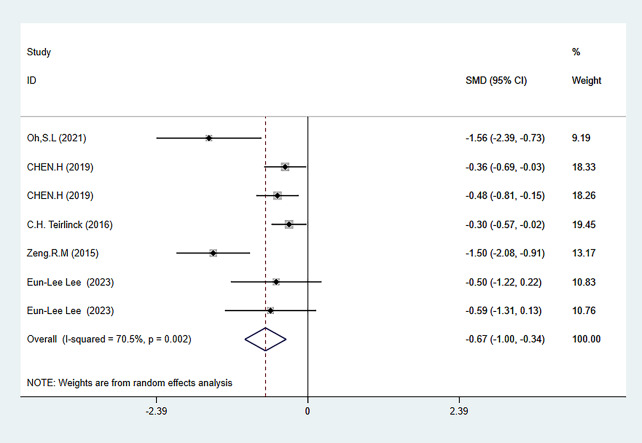
Forest map of meta-analysis of balancing ability

The two secondary outcome indicators of this meta-analysis were activity level (ADL) (evaluated using GS and 6MWT combined) and balance level (evaluated using TUG and FTSST combined). The results showed that HBE can significantly improve the balance ability ADL of KOA and HipOA patients [SMD = 0.51, 95% CI (0.19, 0.82); *P* = 0.002], [SMD=-0.67, 95% CI (-1.00, -0.34); *P* = 0.001]. The results of secondary outcome measures are shown in Figs. 6 and 7, and Table 2. The grade rating of the secondary outcome measure is shown in Table 3.

**Table 3:** Grade rating for outcome indicators

Primary outcome measures	Grade
pain	Moderate
stiffness	Low
function	Moderate
Secondary outcome measures	
balance	Low
ADL	Moderate

##### Mobility: ADL

ADL was evaluated using a combination of GS and 6MWT tests. A total of 7 articles reported relevant tests [26, 29, 32–35, 38] involving 568 individuals, including 317 in the HBE group and 251 in the control group. The heterogeneity test showed I^2^ = 66.5%. Therefore, a random effects model was used to analyze the included studies, and the analysis results showed that HBE had a better effect on improving the activity ability of KOA and HipOA patients than the control group [SMD = 0.51, 95% CI (0.19, 0.82); *P* = 0.002] (Fig. 6).

##### Balance ability

The balance ability was evaluated through a combination of TUG and FTSST tests. A total of 5 articles reported relevant tests [26, 31, 32, 38, 39], and 608 patients were involved, including 311 in the HBE group and 297 in the control group. The heterogeneity test showed I^2^ = 70.5%. Hence, a random-effects model was used to analyze the included studies. The analysis results showed that HBE was more effective in improving the balance ability of KOA and HipOA patients than the control group [SMD=-0.67, 95% CI (-1.00, -0.34); *P* = 0.001] (Fig. 7).

### Subgroup analysis

Subgroup analysis was conducted based on the location of arthritis to further explore the therapeutic effect of HBE on different types of arthritis. Meanwhile, the sources of heterogeneity were explored through subgroup analysis due to the significant heterogeneity in the results of meta-analysis. According to the comprehensive subgroup analysis results, compared with the control group, HBE can significantly improve the joint function [SMD=-0.91, 95% CI (-1.66, -0.17); *P* = 0.016], balance ability [SMD=-0.58, 95% CI (-0.88, -0.27); *P* = 0.001], and ADL [SMD = 0.57, 95% CI (0.04,1.11); *P* = 0.036] in KOA patients, joint function in HipOA patients [SMD=-0.92, 95% CI (-1.45, -0.38); *P* = 0.001], and ADL in patients with comorbidities of KOA and HipOA [SMD = 0.44, 95% CI (0.14, 0.74); *P* = 0.004]. There was no significant difference in other outcomes compared with the control group. In addition, according to the I^2^ values of each subgroup analysis, the type of arthritis in different parts may be the reason for the high heterogeneity in the meta-analysis results of pain, balance ability, and ADL, but it is not the reason for the high heterogeneity in the meta-analysis results of joint stiffness and joint function. The results of subgroup analysis are shown in Supplementary Figs. 1–5 and Table 4.

**Table 4:** Summary of subgroup analysis

Measurements	Number of included articles	Subgroup	I² value (%)	SMD/WMD (95% CI)	*P*-value
primary outcome measures					
pain	10	KOA	61.3	-0.44(-0.71, -0.18)	0.001
	2	HipOA	37.8	-0.37(-0.72, -0.02)	0.041
	2	KOA&HipOA	70.4	-0.17(-0.55, 0.21)	0.384
stiffness	4	KOA	92	-0.75(-1.66,0.17)	0.109
	1	KOA&HipOA	None	-1.10(-2.21, 0.01)	0.053
function	6	KOA	92.5	-0.91 (-1.66, -0.17)	0.016
	1	HipOA	None	-0.92(-1.45, -0.38)	0.001
	2	KOA&HipOA	85.8	0.04 (-0.44, 0.52)	0.864
Secondary outcome measures					
balance	3	KOA	43.4	-0.58 (-0.88, -0.27)	0.001
	2	HipOA	92.5	-0.87 (-2.04, 0.30)	0.147
ADL	3	KOA	54.2	0.57 (0.04, 1.11)	0.036
	2	HipOA	91.9	0.57 (-0.58, 1.72)	0.334
	2	KOA&HipOA	0	0.44 (0.14, 0.74)	0.004

### Sensitivity analysis

Sensitivity analysis was conducted on the data results of pain, joint stiffness, joint function, ADL, and balance ability in 16 articles to determine the stability of the comprehensive results. The results showed that the circles representing each study were within the range of the original confidence interval effect values, indicating that the analysis results were relatively stable, as shown in Supplementary Fig. 6.

### Publication bias evaluation

**Fig. 8:**
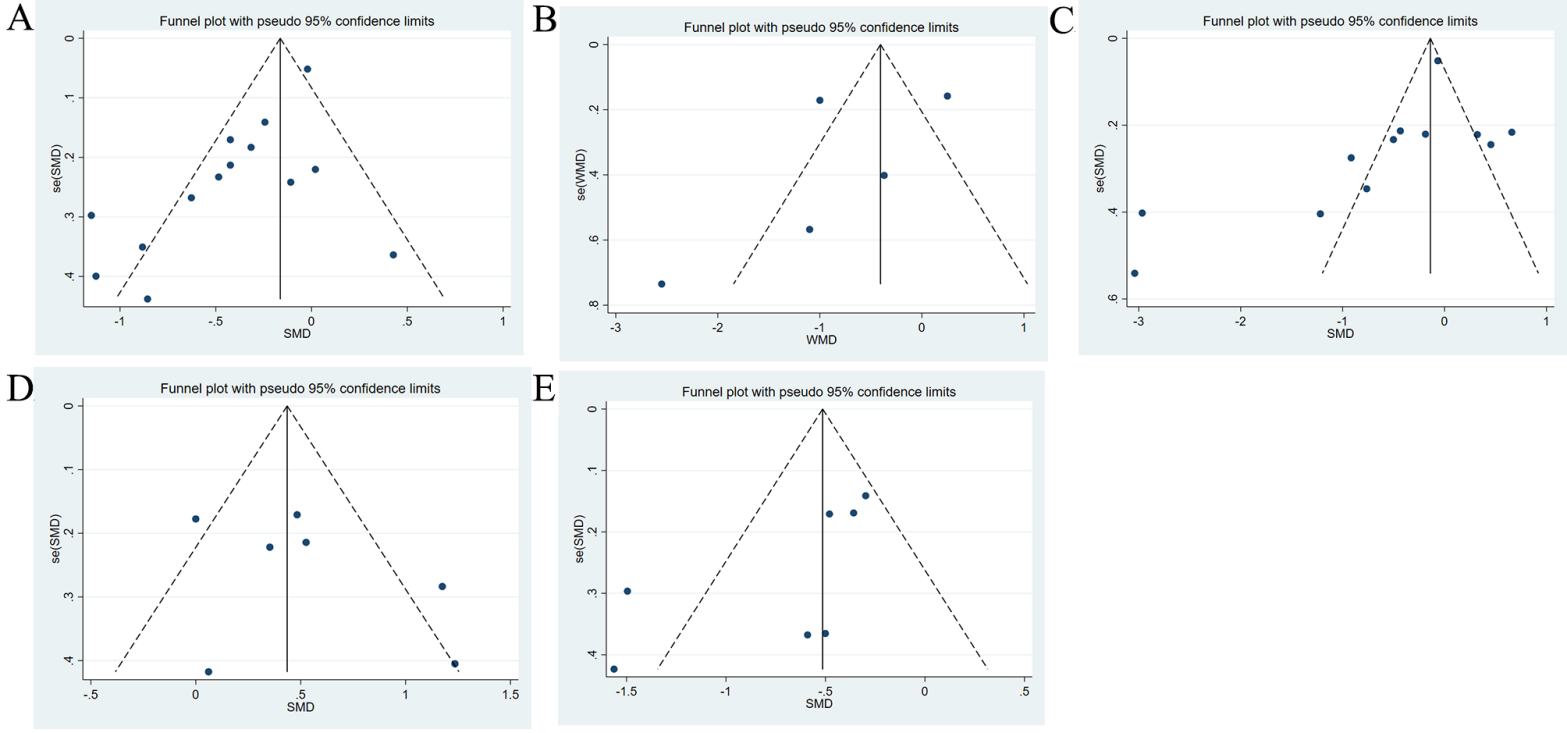
Funnel diagram. (**A**) Pain; (**B**) Joint stiffness; (**C**) Joint function; (**D**) ADL; (**E**) Balance ability

A funnel plot was used to evaluate publication bias for the outcomes. Subsequently, Egger’s test was further used to statistically test the publication bias. The results showed that the Egger’s test P-values for joint stiffness (P Egger = 0.127), joint function (P Egger = 0.096), ADL (P Egger = 0.383), and balance ability (P Egger = 0.144) were all > 0.05, indicating that there may be no publication bias. The P value of Egger’s test for pain was less than 0.05 (P Egger = 0.003), and no study was added after two iterations of the trim and fill method. The results showed no difference from the original results, indicating that there may be publication bias. However, the publication bias has little effect on the results of this study. The funnel plot is shown in Fig. 8A-E, and the Egger’s test results are shown in Supplementary Fig. 7.

## Discussion

For patients with KOA and HipOA, HBE is a cost-effective and easily-promoted mode of physical activity, which is convenient and simple to perform with no use or minimal use of medical equipment. It can reduce psychological fear in patients and alleviate the economic burden on those with transportation difficulties or financial distress to visit physical therapists. Several studies have demonstrated the effectiveness of HBE in relieving joint pain, improving physical function, and enhancing quality of life [42, 43]. Chronic pain patients often tend to have a vicious cycle of physical inactivity, prolonged sitting, and disability exacerbation [44]. For patients suffering from long-term OA, pain may become a source of exercise phobia, and muscle strength may directly or indirectly influence their physical activities [45]. Studies have found no significant correlation between HBE and central sensitization or pain intensity, and exercise intensity does not induce more adverse reactions [46–48]. Low educational level has been identified as an important factor contributing to catastrophic pain and exercise phobia in OA patients [49], highlighting the need for healthcare providers to pay attention to psychological behavior induction and pain neuroscience education to eliminate patient fear of exercise. The efficacy of HBE is also related to patient compliance. Future research on behavioral interventions is needed to increase long-term exercise adherence. Patient compliance is influenced by factors such as supervision, family support, emotional involvement, and trust in physical therapists [50, 51], which can be improved through self-management plans, personalized programs, monitoring and feedback, cognitive-behavioral techniques, and other interventions [52]. However, some studies have shown that there is no specificity in treatment outcomes between supervised and unsupervised HBE, and compared to short-term supervised physical therapy, long-term HBE programs have better long-term outcomes for limb function [30, 53–55]. Therefore, how to improve patient motivation and provide HBE programs that are easy to adhere to in the long-term is a problem that needs further attention. This study has several imitations. First, the intervention methods were relatively single, and we did not take into account comprehensive factors such as self-management, supervised exercise, and health education that may affect patient compliance and final treatment outcomes. Further analysis can be conducted to analyze the efficacy of HBE for patients with severe pain after using combined drug or physical interventions. Second, our outcome indicators mainly focused on pain, and somatic function, with a lack of evaluation of psychological health, life quality. Third, the HBE programs should be personalized according to the different locations of joint wear and muscle and ligament injuries in patients. This study only partially summarizes the therapeutic effects of HBE, and more high-quality, large-scale studies are needed to explore the true efficacy of HBE.

## Conclusion

The present study shows that HBE can significantly improve pain, joint function, balance ability, and mobility in patients with KOA and HipOA. Due to the limitations of this study, further clinical data and high-quality research are needed in the future to confirm our conclusions.

### Electronic supplementary material

Below is the link to the electronic supplementary material.

Supplementary Material 1

Supplementary Material 2

## Data Availability

All data generated or analyzed during this study are included in this published article (and its Supplementary Information files).

## Abbreviations

6MWT: Six-minute walk test
ACR: American College of Rheumatology
ADL: Activity level
ARA: American Rheumatology Association
CI: Confidence interval
FTSST: Five-times sit-to-stand test
GS: Gait speed
HBE: Home-based exercise
HipOA: Hip osteoarthritis
KL scale: Kellgren Lawrence classification
KOA: Knee osteoarthritis
MD: Mean difference
OARSI: Osteoarthritis Research Society International
SMD: Standardized mean difference
TCS: Timed chair stand
TUG: Timed up and go test
VAS: Visual Analogue Scale
WOMAC: Western Ontario and McMaster Universities Osteoarthritis Index
RCT: Randomized controlled trial
