# Supplementary material for: Efficacy of home-based exercise in the treatment of pain and disability at the hip and knee in patients with osteoarthritis: a systematic review and meta-analysis
Source: BMC Musculoskelet Disord. 2024 Jun 26;25:499. doi: 10.1186/s12891-024-07585-w (PMC11201901; doi:10.1186/s12891-024-07585-w)
Supplement: Supplementary file 2 — Supplementary Material 2 [file 12891_2024_7585_MOESM2_ESM.docx]

**Supplementary Table 1** Retrieval Strategy.

| **Database** | **Search Strategies** | **Number of Articles** |
| --- | --- | --- |
| PubMed | ((((residential[Title/Abstract]) OR (home[Title/Abstract])) OR (home-based[Title/Abstract])) AND (("Exercise"[Mesh]) OR ((((((((((((((((((((((((((Exercise[Title/Abstract]) OR (Exercises[Title/Abstract])) OR (Physical Activity[Title/Abstract])) OR (Activities, Physical[Title/Abstract])) OR (Activity, Physical[Title/Abstract])) OR (Physical Activities[Title/Abstract])) OR (Exercise, Physical[Title/Abstract])) OR (Exercises, Physical[Title/Abstract])) OR (Physical Exercise[Title/Abstract])) OR (Physical Exercises[Title/Abstract])) OR (Acute Exercise[Title/Abstract])) OR (Acute Exercises[Title/Abstract])) OR (Exercise, Acute[Title/Abstract])) OR (Exercises, Acute[Title/Abstract])) OR (Exercise, Isometric[Title/Abstract])) OR (Exercises, Isometric[Title/Abstract])) OR (Isometric Exercises[Title/Abstract])) OR (Isometric Exercise[Title/Abstract])) OR (Exercise, Aerobic[Title/Abstract])) OR (Aerobic Exercise[Title/Abstract])) OR (Aerobic Exercises[Title/Abstract])) OR (Exercises, Aerobic[Title/Abstract])) OR (Exercise Training[Title/Abstract])) OR (Exercise Trainings[Title/Abstract])) OR (Training, Exercise[Title/Abstract])) OR (Trainings, Exercise[Title/Abstract])))) AND ((("Osteoarthritis, Knee"[Mesh]) OR ((((((((((((((((((((Osteoarthritis, Knee[Title/Abstract]) OR (Knee Osteoarthritides[Title/Abstract])) OR (Knee Osteoarthritis[Title/Abstract])) OR (Osteoarthritis of Knee[Title/Abstract])) OR (Osteoarthritis of the Knee[Title/Abstract])) OR (Gonitis[Title/Abstract])) OR (Gonarthritis[Title/Abstract])) OR (Knee Arthritis[Title/Abstract])) OR (knee osteoarthritis[Title/Abstract])) OR (arthrosis, knee[Title/Abstract])) OR (femorotibial arthrosis[Title/Abstract])) OR (gonarthrosis[Title/Abstract])) OR (knee arthrosis[Title/Abstract])) OR (knee joint arthrosis[Title/Abstract])) OR (knee joint osteoarthritis[Title/Abstract])) OR (knee osteo-arthritis[Title/Abstract])) OR (knee osteo-arthrosis[Title/Abstract])) OR (knee osteoarthrosis[Title/Abstract])) OR (osteoarthritis, knee[Title/Abstract])) OR (osteoarthrosis, knee[Title/Abstract]))) OR (("Osteoarthritis, Hip"[Mesh]) OR (((((((((((((((((Osteoarthritis, Hip[Title/Abstract]) OR (Hip Osteoarthritis[Title/Abstract])) OR (Osteoarthritis Of Hip[Title/Abstract])) OR (Osteoarthritis Of Hips[Title/Abstract])) OR (Coxarthrosis[Title/Abstract])) OR (Coxarthroses[Title/Abstract])) OR (Osteoarthritis of the Hip[Title/Abstract])) OR (Hip Osteoarthritis[Title/Abstract])) OR (arthrosis, hip[Title/Abstract])) OR (arthrosis, hip joint[Title/Abstract])) OR (Coxartherosis[Title/Abstract])) OR (hip arthrosis[Title/Abstract])) OR (hip joint arthrosis[Title/Abstract])) OR (hip osteo-arthritis[Title/Abstract])) OR (hip osteo-arthrosis[Title/Abstract])) OR (malum coxae senilis[Title/Abstract])) OR (osteoarthritis, hip[Title/Abstract])))) | 503 |
| Web of Science | (Osteoarthritis, Knee (Topic) or Knee Osteoarthritides (Topic) or Knee Osteoarthritis (Topic) or Osteoarthritis of Knee (Topic) or Osteoarthritis of the Knee (Topic) or Gonitis (Topic) or Gonarthritis (Topic) or Knee Arthritis (Topic) or Knee Arthritis (Topic) or arthrosis, knee (Topic) or femorotibial arthrosis (Topic) or gonarthrosis (Topic) or knee arthrosis (Topic) or knee joint arthrosis (Topic) or knee joint osteoarthritis (Topic) or knee osteo-arthritis (Topic) or knee osteo-arthrosis (Topic) or knee osteoarthrosis (Topic) or osteoarthritis, knee (Topic) or osteoarthrosis, knee (Topic) ) or (Osteoarthritis, Hip (Topic) or Hip Osteoarthritis (Topic) or Osteoarthritis Of Hip (Topic) or Osteoarthritis Of Hips (Topic) or Coxarthrosis (Topic) or Coxarthroses (Topic) or Osteoarthritis of the Hip (Topic) or Hip Osteoarthritis (Topic) or arthrosis, hip (Topic) or arthrosis, hip joint (Topic) or Coxartherosis (Topic) or hip arthrosis (Topic) or hip joint arthrosis (Topic) or hip osteo-arthritis (Topic) or hip osteo-arthrosis (Topic) or malum coxae senilis (Topic) or osteoarthritis, hip (Topic)) and (Exercise (Topic) or Exercises (Topic) or Physical Activity (Topic) or Activities, Physical (Topic) or Activity, Physical (Topic) or Physical Activities (Topic) or Exercise, Physical (Topic) or Exercises, Physical (Topic) or Physical Exercise (Topic) or Physical Exercises (Topic) or Acute Exercise (Topic) or Acute Exercises (Topic) or Exercise, Acute (Topic) or Exercises, Acute (Topic) or Exercise, Isometric (Topic) or Exercises, Isometric (Topic) or Isometric Exercises (Topic) or Isometric Exercise (Topic) or Exercise, Aerobic (Topic) or Aerobic Exercise (Topic) or Aerobic Exercises (Topic) or Exercises, Aerobic (Topic) or Exercise Training (Topic) or Exercise Trainings (Topic) or Training, Exercise (Topic) or Trainings, Exercise (Topic)) and (residential (Topic) or home (Topic) or home-based (Topic)) | 1255 |
| Cochrane Library | ((Osteoarthritis, Knee)MeSH or ((((((Osteoarthritis, Knee):ti,ab,kw OR (Knee Osteoarthritides):ti,ab,kw OR (Knee Osteoarthritis):ti,ab,kw OR (Osteoarthritis of Knee):ti,ab,kw OR (Osteoarthritis of the Knee):ti,ab,kw) or ((((((Gonitis):ti,ab,kw OR (Gonarthritis):ti,ab,kw OR (Knee Arthritis):ti,ab,kw OR (knee osteoarthritis):ti,ab,kw OR (arthrosis, knee):ti,ab,kw) OR ((Osteoarthritis, Hip)MeSH or(((((((((((((((( (Osteoarthritis, Hip):ti,ab,kw OR (Hip Osteoarthritis):ti,ab,kw OR (Osteoarthritis Of Hip):ti,ab,kw OR (Osteoarthritis Of Hips):ti,ab,kw OR (Coxarthrosis):ti,ab,kw OR (Coxarthroses):ti,ab,kw OR (Osteoarthritis of the Hip):ti,ab,kw OR (Hip Osteoarthritis):ti,ab,kw OR (arthrosis, hip):ti,ab,kw OR (arthrosis, hip joint):ti,ab,kw OR (Coxartherosis):ti,ab,kw OR (hip arthrosis):ti,ab,kw OR (hip joint arthrosis):ti,ab,kw OR (hip osteo-arthritis):ti,ab,kw OR (hip osteo-arthrosis):ti,ab,kw OR (malum coxae senilis):ti,ab,kw OR (osteoarthritis, hip):ti,ab,kw) AND ((Exercise)MeSH or ((((((((((((((((((((((((((Exercise):ti,ab,kw OR (Exercises):ti,ab,kw OR (Physical Activity):ti,ab,kw OR (Activities, Physical):ti,ab,kw OR (Activity, Physical):ti,ab,kw OR (Physical Activities):ti,ab,kw OR (Exercise, Physical):ti,ab,kw OR (Exercises, Physical):ti,ab,kw OR (Physical Exercise):ti,ab,kw OR (Physical Exercises):ti,ab,kw OR (Acute Exercise):ti,ab,kw OR (Acute Exercises):ti,ab,kw OR (Exercise, Acute):ti,ab,kw OR (Exercises, Acute):ti,ab,kw OR (Exercise, Isometric):ti,ab,kw OR (Exercises, Isometric):ti,ab,kw OR (Isometric Exercises):ti,ab,kw OR (Isometric Exercise):ti,ab,kw OR (Exercise, Aerobic):ti,ab,kw OR (Aerobic Exercise):ti,ab,kw OR (Aerobic Exercises):ti,ab,kw OR (Exercises, Aerobic):ti,ab,kw OR (Exercise Training):ti,ab,kw OR (Exercise Trainings):ti,ab,kw OR (Training, Exercise):ti,ab,kw OR (Trainings, Exercise):ti,ab,kw) AND ((((residential):ti,ab,kw OR (home):ti,ab,kw OR (home-based):ti,ab,kw) | 172 |
| EMBASE | ((('knee osteoarthritis'/exp) or(((((((((((((((((( 'knee osteoarthritides':ti,ab,kw) OR ('osteoarthritis of knee':ti,ab,kw) OR( 'osteoarthritis of the knee':ti,ab,kw) OR (gonitis:ti,ab,kw ) OR( gonarthritis:ti,ab,kw) OR ('knee arthritis':ti,ab,kw) OR( 'knee osteoarthritis':ti,ab,kw) OR ('arthrosis, knee':ti,ab,kw) OR( 'femorotibial arthrosis':ti,ab,kw) OR (gonarthrosis:ti,ab,kw) OR( 'knee arthrosis':ti,ab,kw) OR( 'knee joint arthrosis':ti,ab,kw) OR ('knee joint osteoarthritis':ti,ab,kw) OR( 'knee osteo-arthritis':ti,ab,kw) OR( 'knee osteo-arthrosis':ti,ab,kw) OR ('knee osteoarthrosis':ti,ab,kw) OR ('osteoarthritis, knee':ti,ab,kw) OR ('osteoarthrosis, knee':ti,ab,kw) )) )OR ((('hip osteoarthritis'/exp) or(((((((((((((((( 'osteoarthritis of hip':ti,ab,kw) OR( 'osteoarthritis of hips':ti,ab,kw )OR( coxarthrosis:ti,ab,kw) OR (coxarthroses:ti,ab,kw) OR( 'osteoarthritis of the hip':ti,ab,kw) OR ('hip osteoarthritis':ti,ab,kw) OR( 'arthrosis, hip':ti,ab,kw) OR ('arthrosis, hip joint':ti,ab,kw) OR( coxartherosis:ti,ab,kw) OR( 'hip arthrosis':ti,ab,kw) OR ('hip joint arthrosis':ti,ab,kw )OR( 'hip osteo-arthritis':ti,ab,kw) OR ('hip osteo-arthrosis':ti,ab,kw) OR( 'malum coxae senilis':ti,ab,kw )OR( 'osteoarthritis, hip':ti,ab,kw))) )AND(((((((((((((((((((((((((((((((( exercise:ti,ab,kw )OR ( exercises:ti,ab,kw )OR( 'physical activity':ti,ab,kw )OR ( 'activities, physical':ti,ab,kw )OR ( 'activity, physical':ti,ab,kw )OR ( 'physical activities':ti,ab,kw) OR ( 'exercise, physical':ti,ab,kw )OR ( 'exercises, physical':ti,ab,kw )OR( 'physical exercise':ti,ab,kw )OR( 'physical exercises':ti,ab,kw )OR ( 'acute exercise':ti,ab,kw) OR ( 'acute exercises':ti,ab,kw )OR ( 'exercise, acute':ti,ab,kw) OR( 'exercises, acute':ti,ab,kw) OR ( 'exercise, isometric':ti,ab,kw )OR ( 'exercises, isometric':ti,ab,kw )OR ( 'isometric exercises':ti,ab,kw )OR ( 'isometric exercise':ti,ab,kw )OR( 'exercise, aerobic':ti,ab,kw )OR ( 'aerobic exercise':ti,ab,kw) OR( 'aerobic exercises':ti,ab,kw )OR ( 'exercises, aerobic':ti,ab,kw )OR( 'exercise training':ti,ab,kw) OR ( 'exercise trainings':ti,ab,kw )OR( 'training, exercise':ti,ab,kw )OR ( 'trainings, exercise':ti,ab,kw)) AND ((((residential:ti,ab,kw) OR (home:ti,ab,kw) OR( 'home based':ti,ab,kw)) | 610 |
| **Total Number of Articles** | | **2540** |

**
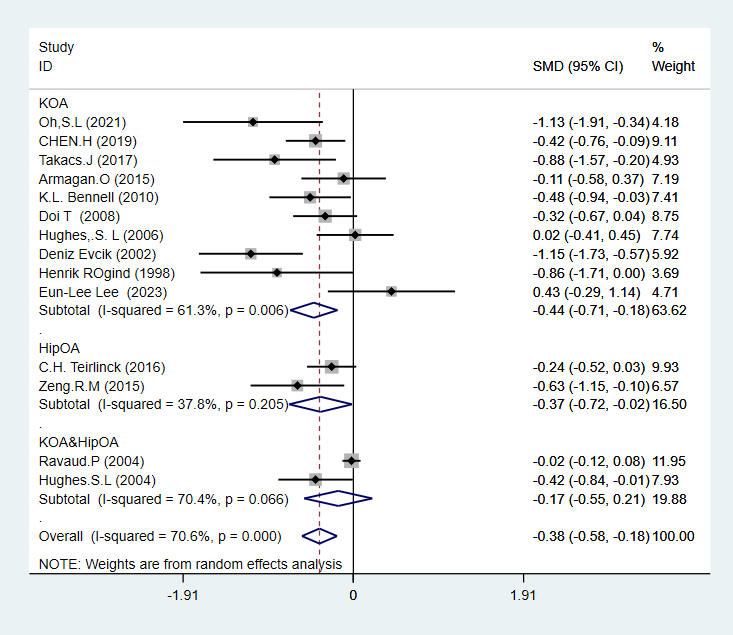
**

**Supplementary Figure 1** Subgroup analysis of pain.

**
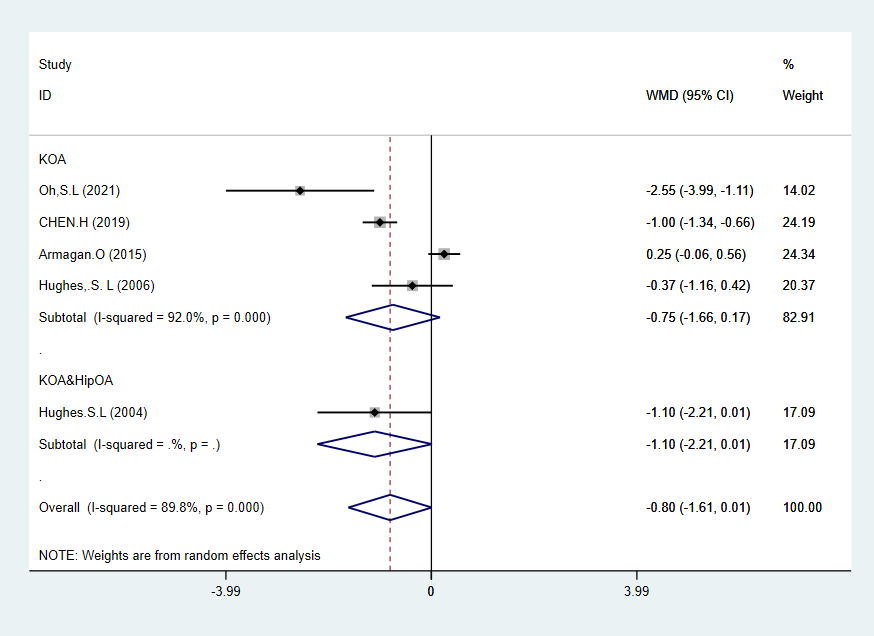
**

**Supplementary Figure 2** Subgroup analysis of joint stiffness


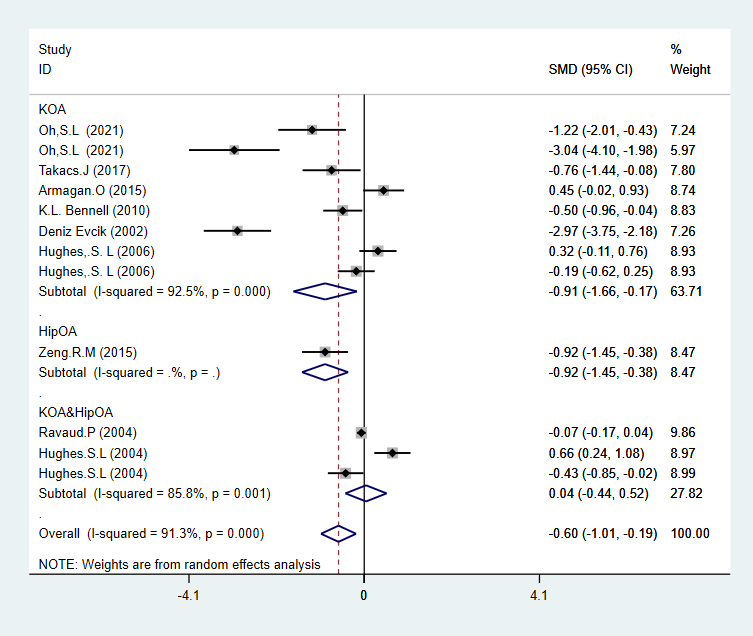
**Supplementary Figure 3** Subgroup analysis of joint function.

**
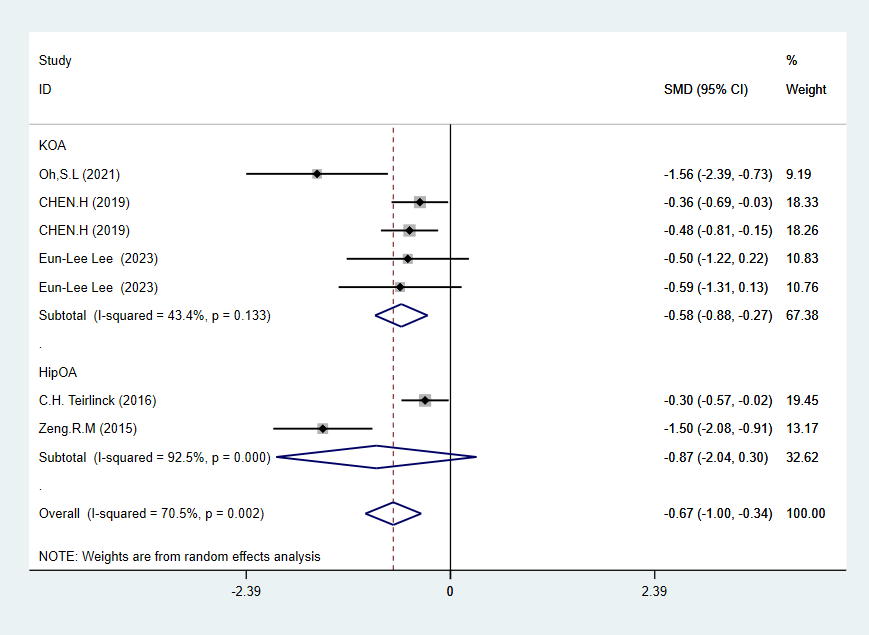
**

**Supplementary Figure 4** Subgroup analysis of balance ability.

**
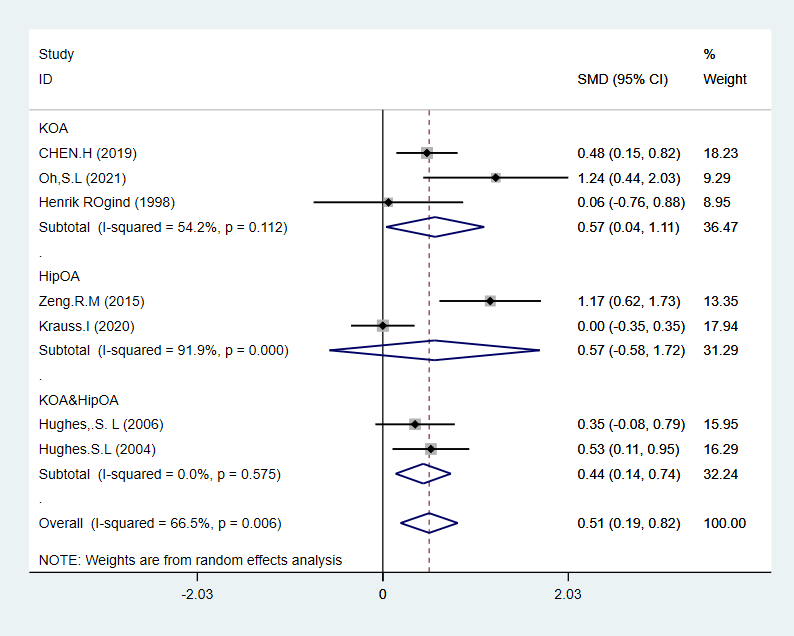
**

**Supplementary Figure 5** Subgroup analysis of activity ability.

**
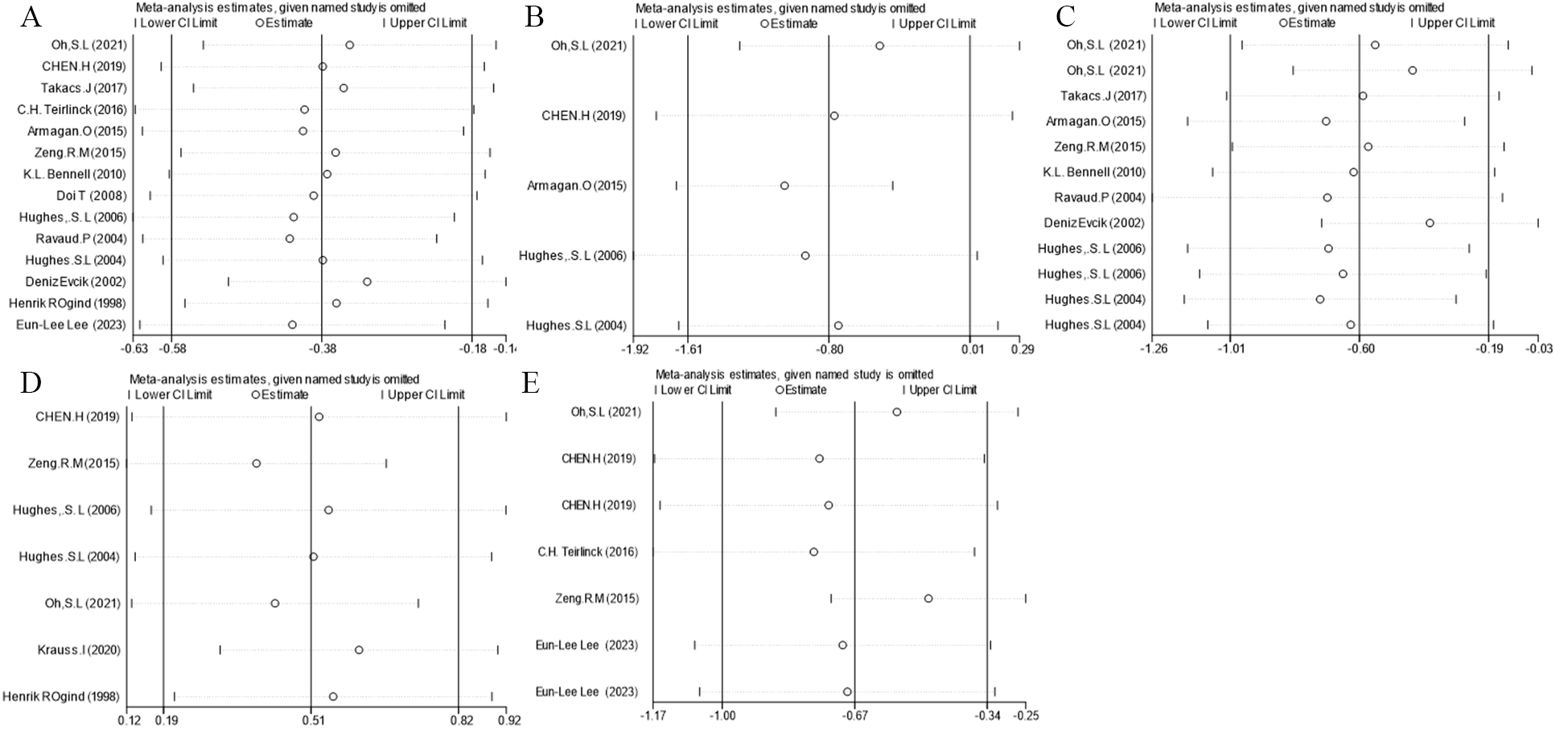
**

**Supplementary Figure 6** Sensitivity analysis. A. Pain; B. Joint stiffness; C. Joint function; D. ADL; E. Equilibrium ability.

**
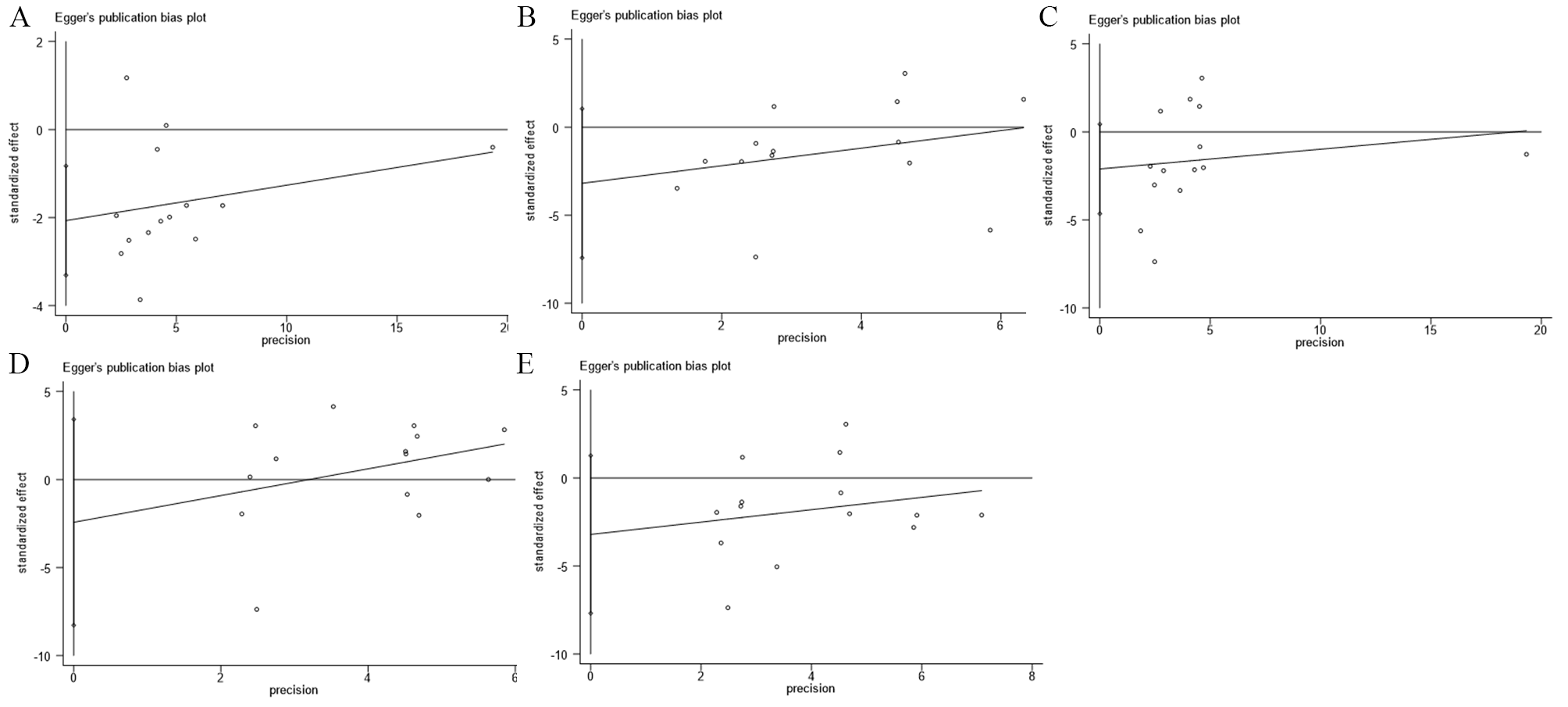
**

**Supplementary Figure 7** Egger’s Test. A. Pain; B. Joint stiffness; C. Joint function; D. ADL; E. Equilibrium ability.
